# Use of Tethered Enzymes as a Platform Technology for Rapid Analyte Detection

**DOI:** 10.1371/journal.pone.0142326

**Published:** 2015-11-25

**Authors:** Roy Cohen, James P. Lata, Yurim Lee, Jean C. Cruz Hernández, Nozomi Nishimura, Chris B. Schaffer, Chinatsu Mukai, Jacquelyn L. Nelson, Sharon A. Brangman, Yash Agrawal, Alexander J. Travis

**Affiliations:** 1 Baker Institute for Animal Health, College of Veterinary Medicine, Cornell University, Hungerford Hill Rd., Ithaca, NY 14853, United States of America; 2 Department of Biomedical Engineering, Cornell University, Ithaca, NY 14853, United States of America; 3 Central New York ADAC, SUNY Upstate Medical University, Syracuse, NY 13210, United States of America; 4 New York Presbyterian Hospital-Cornell Campus, Cornell University, Ithaca, NY 10065, United States of America; 5 Atkinson Center for a Sustainable Future, Cornell University, Ithaca, NY 14853, United States of America; Wageningen University, NETHERLANDS

## Abstract

**Background:**

Rapid diagnosis for time-sensitive illnesses such as stroke, cardiac arrest, and septic shock is essential for successful treatment. Much attention has therefore focused on new strategies for rapid and objective diagnosis, such as Point-of-Care Tests (PoCT) for blood biomarkers. Here we use a biomimicry-based approach to demonstrate a new diagnostic platform, based on enzymes tethered to nanoparticles (NPs). As proof of principle, we use oriented immobilization of pyruvate kinase (PK) and luciferase (Luc) on silica NPs to achieve rapid and sensitive detection of neuron-specific enolase (NSE), a clinically relevant biomarker for multiple diseases ranging from acute brain injuries to lung cancer. We hypothesize that an approach capitalizing on the speed and catalytic nature of enzymatic reactions would enable fast and sensitive biomarker detection, suitable for PoCT devices.

**Methods and findings:**

We performed in-vitro, animal model, and human subject studies. First, the efficiency of coupled enzyme activities when tethered to NPs versus when in solution was tested, demonstrating a highly sensitive and rapid detection of physiological and pathological concentrations of NSE. Next, in rat stroke models the enzyme-based assay was able in minutes to show a statistically significant increase in NSE levels in samples taken 1 hour before and 0, 1, 3 and 6 hours after occlusion of the distal middle cerebral artery. Finally, using the tethered enzyme assay for detection of NSE in samples from 20 geriatric human patients, we show that our data match well (r = 0.815) with the current gold standard for biomarker detection, ELISA—with a major difference being that we achieve detection in 10 minutes as opposed to the several hours required for traditional ELISA.

**Conclusions:**

Oriented enzyme immobilization conferred more efficient coupled activity, and thus higher assay sensitivity, than non-tethered enzymes. Together, our findings provide proof of concept for using oriented immobilization of active enzymes on NPs as the basis for a highly rapid and sensitive biomarker detection platform. This addresses a key challenge in developing a PoCT platform for time sensitive and difficult to diagnose pathologies.

## Introduction

There is pressing need for quick and objective diagnostic technologies for both time sensitive and difficult to diagnose pathologies. Much attention has therefore focused on the identification of disease-specific peripheral biomarkers, and use of new technologies to improve antibody-based detection capabilities. This interest is well justified because of the enormous medical, social and economic impacts of these diseases, in both developed and developing countries. This manuscript presents data about an alternative technological approach to biomarker detection—the use of enzymes tethered to NPs. For proof of principle, we’ll focus on a single biomarker, NSE, that has been suggested to have value for diagnosing various pathologies (particularly those involving the central nervous system). No single biomarker is known to be sensitive and specific enough to diagnose a complex neural disease state. Rather, we present these data as proof of principle for the underlying diagnostic platform suitable for rapid detection of biomarker analytes.

Stroke is one of the best examples of a brain disease that is both hard to diagnose and time sensitive, annually affecting over 15 million people. Of these, 5 million die and an additional 5 million are left permanently disabled [1]. Currently, its diagnosis relies on neurologists to distinguish stroke from mimics using advanced imaging tests (e.g. CT, MRI) in order to determine whether the stroke is ischemic or hemorrhagic. If appropriate, thrombolytic treatment can then be initiated, but is most effective only within a short 3–4 hour window from onset [2]. Remarkably, due to the time constraints for initiating effective treatment, lack of prompt diagnosis for ischemic stroke results in less than 4% of patients in the US receiving thrombolytic therapy [3]. In addition to rapid diagnosis of stroke, there is growing military and civilian need for PoCT devices that provide objective, biomarker-based, diagnostic and prognostic information regarding traumatic brain injuries (TBI/concussion), and neurodegenerative disease [e.g. Alzheimer’s disease (AD), Parkinson’s Disease (PD)]. For these brain injuries, an objective PoCT could lead to interventions beginning early upon the onset of disease as well as provide a platform to monitor disease progression or response to therapy. From a global perspective, the increasing incidence of stroke in developing countries [4], the high cost and limited availability of health resources associated with stroke diagnosis, significantly reduces favorable outcomes [5].

Traditional technologies for blood biomarker diagnostics include ELISA, PCR, and mass spectrometry (MS). However, these technologies are limited to laboratory use because they require sample preparation, and use of sophisticated instruments and highly trained technicians. Moreover, they are time and labor intensive, and are expensive [6]. Much of the research on detection of blood biomarkers has focused on antibody-based methods. Although there has been considerable progress in the field, such as the introduction of lateral flow based assays, constraints due to the speed of antibody-antigen interactions, low sensitivity, semi-quantitative results and the complexity of associated detection instrumentation have thus far prevented such antibody capture methods from becoming the basis for a field-capable PoCT device to detect brain injuries [6].

Over the last few decades, a large number of molecules have been studied for their potential usefulness as blood-borne biomarkers. Although no single biomarker will likely provide a definitive diagnosis of any disease, the glycolytic enzyme, NSE, is released from damaged neurons and has been suggested to be valuable for the diagnosis of various brain injuries [7] [8] [9]. Evidence also supports its usefulness as a prognostic indicator in predicting neurological outcomes of post-cardiac arrest [10] and mild TBI [11]. NSE has been suggested to be useful in distinguishing stroke from mimics, an important first step in expediting the diagnostic process [7]. In addition, it has been suggested that changes in serum NSE levels may indicate changes in brain morphology in AD [12]. Overall, changes in plasma NSE levels are of clinical significance to several of the major brain injuries, making it an excellent first candidate to test a new diagnostic platform technology.

As an alternative to antibody capture, fluid phase enzymatic reactions have been used to detect plasma NSE by means of its enzymatic activity [13] [14]; however, in the 30 years since these assays were first described, to our knowledge, no related PoCT technologies have been reported. Semi-solid phase bioluminescence detection of NSE through its binding to immunobeads enabled high sensitivity detection [15]. However, this method required long serum-bead incubation times and did not tether the downstream enzymes (i.e. PK and Luc). Tethering enzymes has several advantages for a PoCT, such as concentrating the detection reaction, spatially localizing the readout, and potentially improving shelf life. The principle obstacle lies in maintaining enzymatic function when immobilizing enzymes. Immobilization often interferes with substrate binding sites and/or needed conformational changes [16].

To overcome this obstacle, we adopted a strategy of biomimicry, inspired by the tethering of glycolytic enzymes to a cytoskeletal scaffold in the sperm flagellum, where they provide localized energy production [17] [18]. We previously showed that oriented immobilization via genetically-encoded binding domains conferred advantages in specific activity versus either random adsorption or chemically-specific binding [19, 20]. Here, we extend our previous studies [19] [20] in a novel direction, to generate a PoCT platform based on enzymes tethered to nanoparticles via biomimetic oriented immobilization (Tethered Enzyme Technology, TET). Our approach couples production of ATP by PK with activity of firefly luciferase (Luc) to generate a highly rapid and sensitive bioluminescent readout for the detection of NSE. As a tethering scaffold for the PoCT device, we employed SiO_2_ nanoparticles for their many positive attributes: high biocompatibility, low optical absorption, dispersibility, high surface area, and the ability to be integrated into various devices with spatial control. We hypothesized that oriented immobilization of PK and Luc on SiO_2_ NPs could provide the basis for highly rapid, quantitative and sensitive detection of NSE through an enzymatic cascade reaction in a form suitable for use in a PoCT.

## Results

**Fig 1:**
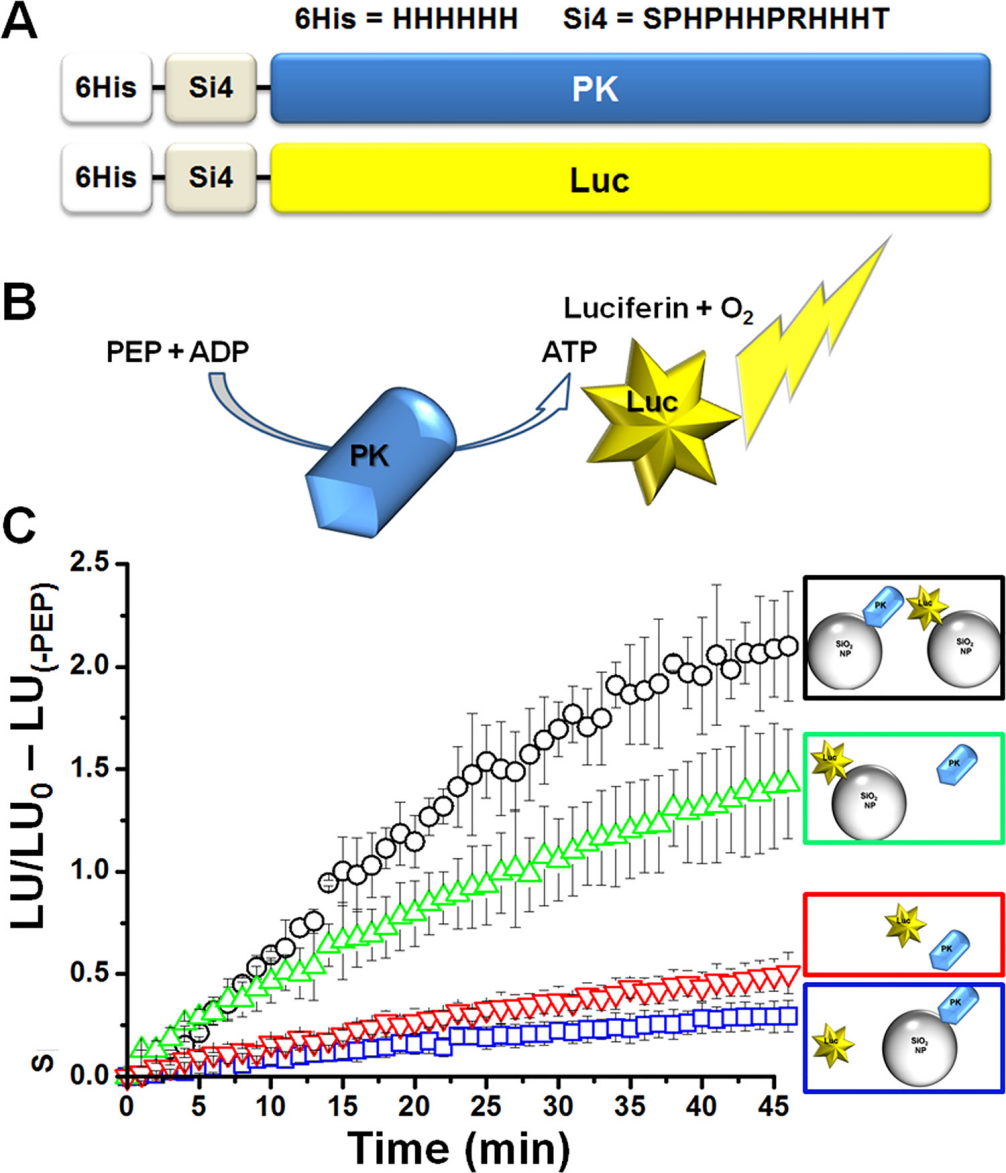
Immobilization of PK and Luc on NPs improves coupled reaction efficiency. **A)** Schematic illustration of the 6XHis-Si tag fusion constructs for PK or Luc. **B)** Schematic representation of the PK-Luc coupled reaction as used in the experiments described in C. **C)** PK and Luc coupled activity was assayed in 4 combinations as indicated by the color coded schematic illustrations: black- (NP-PK) + (NP-Luc), blue- (NP-PK) + (soluble Luc), green- (NP-Luc) + (soluble PK) or red- (soluble PK + soluble Luc). All combinations included equivalent amounts of PK and Luc. Maximal coupled reaction efficiency, as calculated from normalizing each time point data to t_0_ (indicating the ratio of luminescence generated at each time point relative to the luminescence at the beginning of the reaction) and subtraction of the negative control well (no PEP, corresponding to the background luminescence signal), was observed when both PK and Luc were immobilized on NPs (each condition was tested in triplicates; data shown represents 3 individual experiments; AVG±STDEV).

We have previously shown that oriented immobilization of the glycolytic enzymes, TPI and GAPDH, enhances both their individual activities as well as the activity of their coupled sequential reactions in comparison to enzymes tethered via chemically-specific but non-oriented carboxyl-amine binding [20]. To test whether this would hold true for the coupled sequential reactions of PK and the non-glycolytic enzyme Luc, we generated mammalian PK and Luc expression plasmids, each as a fusion protein with two affinity tags, as described in the Materials and Methods section. Each of the constructs included an amino-terminal silica-binding peptide sequence (Si-tag) [21, 22] for immobilization of proteins onto SiO_2_ nanoparticles (Si-NPs) and a 6xHis-tag to be used for protein purification (Fig 1A, see also S1 Fig).

To test our hypothesis that oriented immobilization would impart an improvement on the specific activity of the coupled reaction (Fig 1B), we measured the coupled activities of various combinations of tethered and free PK and Luc (Fig 1C and S2 Fig). Analysis of all four possible combinations revealed a significant increase in the readout signal when Luc was immobilized (green triangles, 3.01±0.2 fold, p<0.001) and an even larger increase upon immobilization of both Luc and PK (black circles, 4.25±0.12 fold, p<0.001). When Luc was in solution (not tethered), the coupled activity of the PK-Luc reaction was reduced, regardless of whether PK was in solution or tethered, suggesting that having Luc in close proximity with the source of ATP production (PK) was crucial for increased coupled reaction efficiency.

To investigate further the source of the improvement in Luc activity when tethered, we compared Luc in-solution versus immobilized. In agreement with previous findings [23], our measurements revealed a 10-fold average reduction in the sensitivity to ATP of immobilized Luc (data not shown). However, Luc tethered on the Si-NPs exhibited extended photon emission dynamics, with a slower decay rate when compared to the soluble protein (S2B Fig). The differences in the enzymatic activity of immobilized Luc might result from a change in diffusion resistance and/or a slowing of kinetics due to conformational constraints. In terms of the PK-Luc coupled reaction, the reduction in Luc sensitivity was most likely compensated for by the prolonged emission kinetics. For example, placing Luc in closer proximity to PK (such as occurs with particle packing) would enhance the efficiency of ATP channeling by reducing the diffusion distance and effective local reaction volume, thereby increasing the effective local concentration of ATP, and enhancing luminescence output. Additional investigations of the impacts of enzyme tethering/proximity can be found as supplemental material (S2 and S4 Figs).

**Fig 2:**
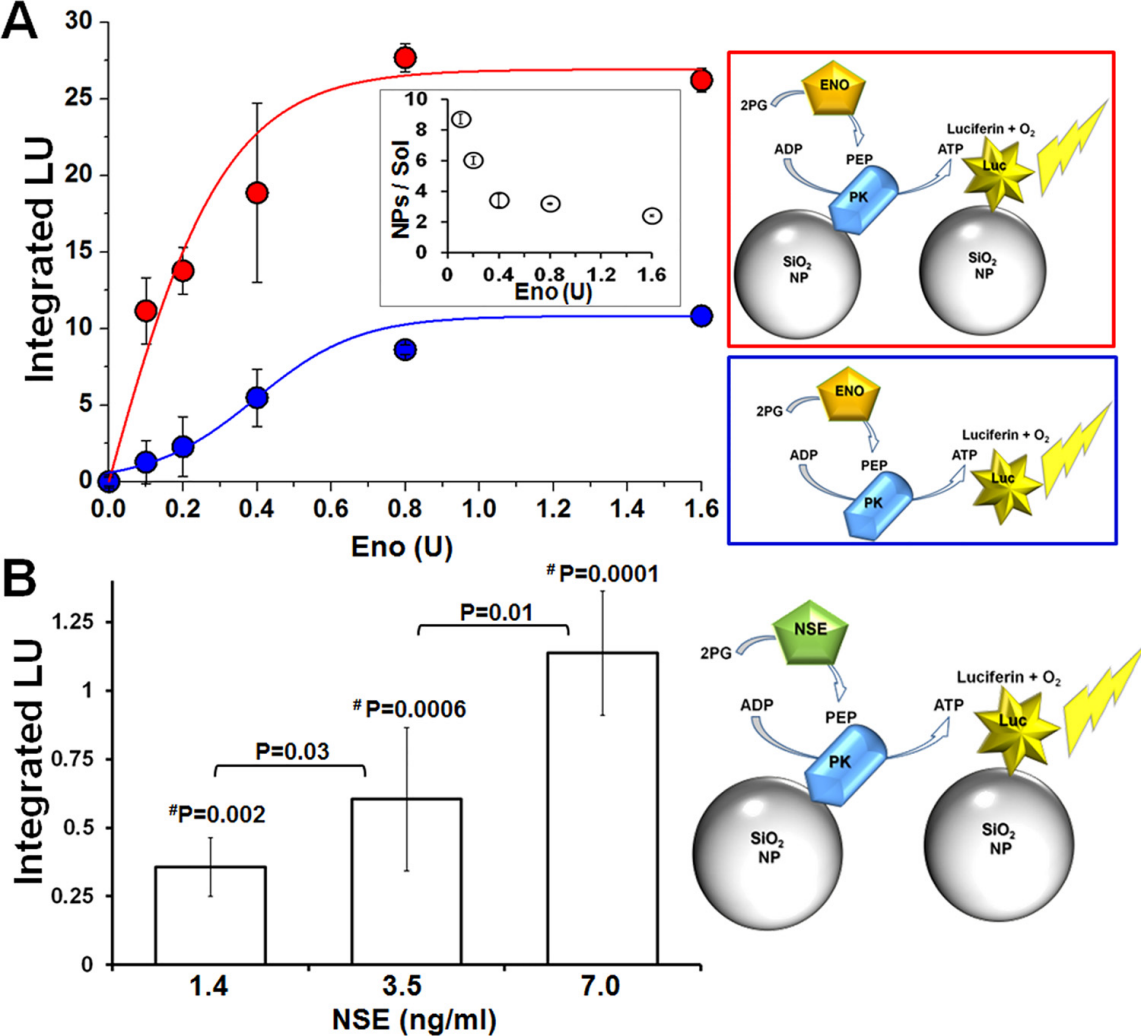
Improved sensitivity for enolase detection via its enzymatic activity when using tethered PK and Luc. **A)** Comparison of the detection sensitivity for enolase activity (Eno) as measured by NP-PK + NP-Luc (red) vs. PK + Luc in solution (blue). Increasing concentrations of Eno were added to immobilized or freely diffusing enzymes in reaction buffer. Luminescence was detected and integrated over 10 minutes at RT and plotted against Eno final unit amount (lines were added to guide the eye). The inset shows the ratio of the enzymes’ activities on NPs versus in solution as a function of units of ENO. Data presented as AVG±STDEV. **B)** NSE detection via PK and Luc coupled activity was performed as indicated in the schematic illustration (right). His-Si-PK and His-Si-Luc were immobilized on 500 nm NPs and mixed with reaction buffer supplemented with increasing concentrations of human NSE. Luminescence readout was normalized to t0 (first read) values and plotted as a function of time. Then, the luminescent signal was integrated for first 10 minutes, and a reading from the zero NSE well was subtracted from the collected signal. The sensitivity of the tethered enzyme assay was about 5 times higher than the reported average physiological NSE blood concentration (8.7 ± 3.9 ng/ml [24]). P values were calculated using student’s t-test for comparisons between NSE concentrations; values marked with ^**#**^P were calculated for significance when compared against 0 ng/ml NSE.

We next hypothesized that the increased efficiency of the immobilized coupled reactions might provide a sensitive and rapid luminescence based assay for detection of the biomarker NSE, through a 3-step coupled reaction as follows–enolase/NSE catalyzes the conversion of 2-phosphoglycerate (2-PG) to phosphoenolpyruvate (PEP), PK in turn converts PEP and ADP to pyruvate and ATP, with the latter being used by Luc to generate a photon of light. We first tested this hypothesis using the non-neuronal isoform α-enolase (ENO). The sensitivity of the tethered enzymes (red, PK and Luc tethered separately to NPs) was significantly higher than that of freely diffusing enzymes (blue) when detecting ENO activity (Fig 2A). In particular, higher sensitivity of the PK-Luc coupled reaction was observed at lower ENO concentrations (Fig 2A inset).

Next, we sought to determine whether the NP-PK and NP-Luc coupled reaction could detect physiologically relevant concentrations of human NSE (< 8.7 ± 3.9 ng/ml [24]). Fig 2B shows a representative dose response experiment where sub-physiological amounts of commercial human NSE were added to the reaction mixture in the presence of NP-PK and NP-Luc (tethered separately). The luminescent signal generated by Luc was integrated over 10 minutes, showing statistically significant differences for each concentration of NSE. These results indicate that using immobilized PK-Luc coupled reactions provides sub-physiological sensitivity for NSE, at concentrations well below typical pathological plasma levels, with rapid detection times.

**Fig 3:**
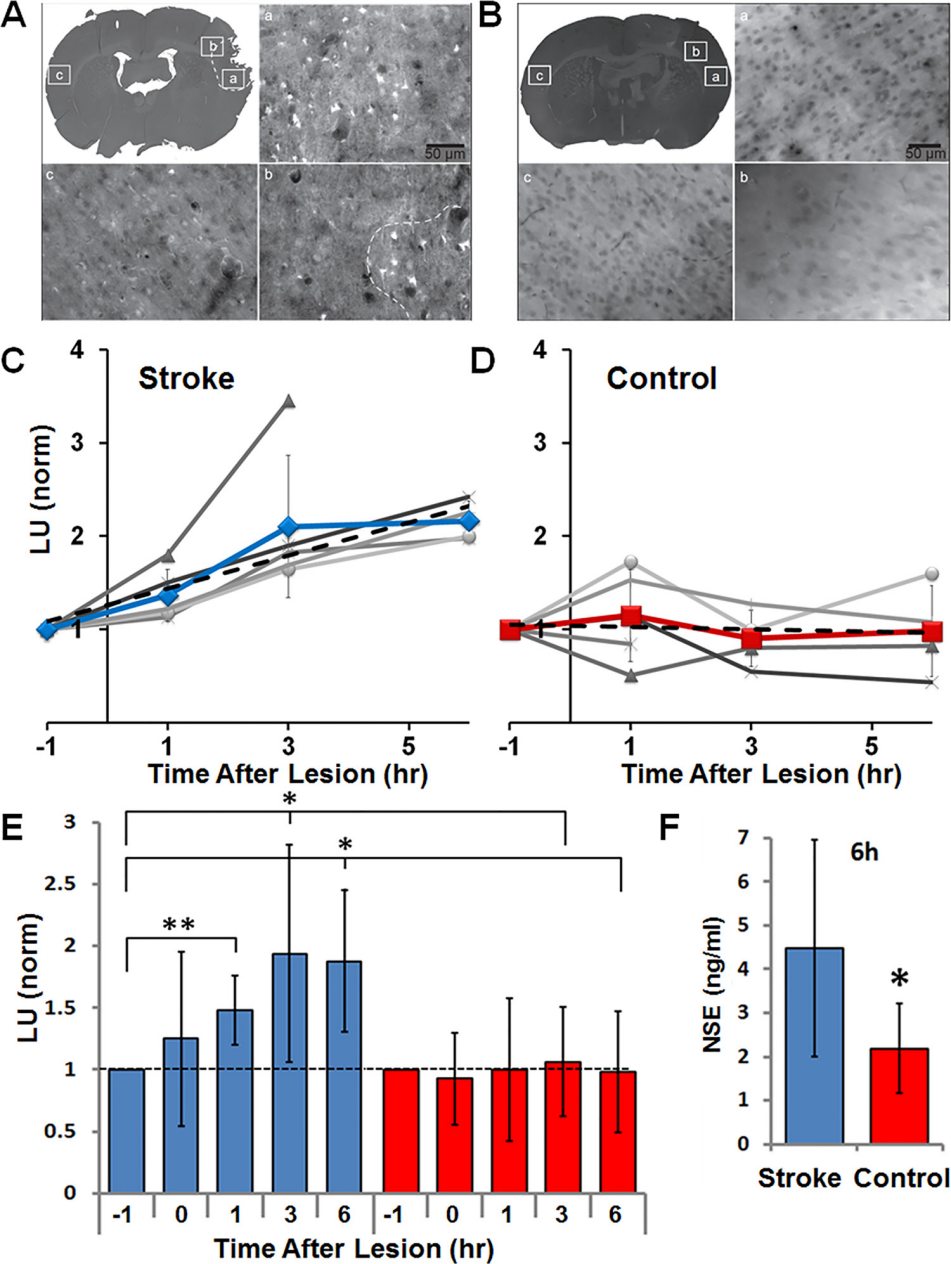
Using NP-PK and NP-Luc for rapid detection of NSE in a rat model for stroke. **A)** Fluoro Jade-C staining for measurement of damaged brain tissue volume. The presence and absence of FJC-labeled degenerative neurons were imaged with epifluorescence under 20x magnification using filters for FITC. (a) Region with abundant FJC staining (bright cells) on lesioned side. (b) Region at the edge of FJC staining. (c) Region that is contralateral to (a) that did not show any FJC staining. Dotted line in B and low magnification inset indicates manually-mapped border between FJC positive and negative areas. **B)** FJC staining in control brain section. (a) and (b) are in area of craniectomy. **C-D)** Individual rat data (C-stroke and D-control) for NSE measurements at each time point (grayscale lines indicate data points for individual animals, line with symbol indicates the mean±standard deviation) as performed by tethered PK and Luc assay, and normalized to time point -1 hour. Plasma samples were collected from stroke induced or control rats pre (-1 hr), and post occlusion (0, 1, 3 and 6 hr). The mean slope of the trend (dashed line) was calculated averaging individual slopes in each group (stroke = 0.26 with 0.95 confidence interval, control = -0.02 with 0.38 confidence interval). **E**) Summary of rat stroke model experiments showing a statistically significant increase in NSE plasma levels in stroke (blue bars) vs. control (red bars) rats as soon as 1 hour post occlusion. **F)** Measurements of NSE in plasma from rats using ELISA showed elevated levels in stroke compared to control rats at the last time point (6 hr post-occlusion). P values– 0.05<*<0.1, 0.01<**<0.05. Data from 10 rats were included in our analysis, with the exclusion of 3 hour and 6 hour time points for one control animal that died (at 2 hour mark), and a 6 hour time point for a stroke animal which died and was found to have a large hemorrhage at the base of the brain.

The improved sensitivity and kinetics of NSE detection with the tethered coupled enzymatic reactions encouraged us to test our biomarker detection assays in the complex environment of blood plasma. For these experiments, we used a rat model for stroke, in the form of a focal stroke that was induced by clotting the distal branches of the middle cerebral artery (MCA). Peripheral blood samples from experimental stroke and sham-operated control rats were taken 1 hour before and 0, 1, 3 and 6 hours after occlusion of distal MCA branches. Luminescence output was integrated over 10 minutes from the NSE-PK-Luc coupled reaction (PK and Luc tethered separately to NPs), but with the active NSE originating from the damaged/dying neurons. Stroke-induced cell death in the rats was confirmed with Fluoro-Jade C staining (see details in Materials and Methods section). The volume of brain with damaged neurons was on average 11.25±4 mm^3^ (n = 4, 3A) in the stroke animals, and 0.002±0.001 mm^3^ in the sham-operated controls (3B n = 4). Integrated luminescence values at each time point were calculated by subtracting a negative control reaction (lacking the NSE substrate, 2-PG) from the test reaction (that included 2-PG). Fig 3E summarizes the data collected from 10 rats (5 stroke and 5 control), showing a statistically significant increase in NSE levels as soon as 1 hour post-occlusion, whereas control rats showed no increase in plasma NSE levels (detailed time point data for each rat are provided in panels 3C and 3D).

Next, we compared results from our tethered enzyme technology (TET) versus quantification of the rat NSE as obtained from a commercial ELISA kit, which is currently the standard detection technology. Consistent with the results from the coupled enzymatic assay at that same time point, the ELISA revealed a 2-fold increase in NSE concentration at 6 hours post stroke (Fig 3F). No elevation was found in non-neuronal isoforms of enolase (NNE) as measured using a NNE ELISA kit (data not shown), demonstrating that the source of enolase activity that we measured was indeed NSE. Importantly, while the measurements of NSE concentration using the “gold standard” ELISA assays took over 3 hours, the TET based assay provided results within 10 minutes.

**Fig 4:**
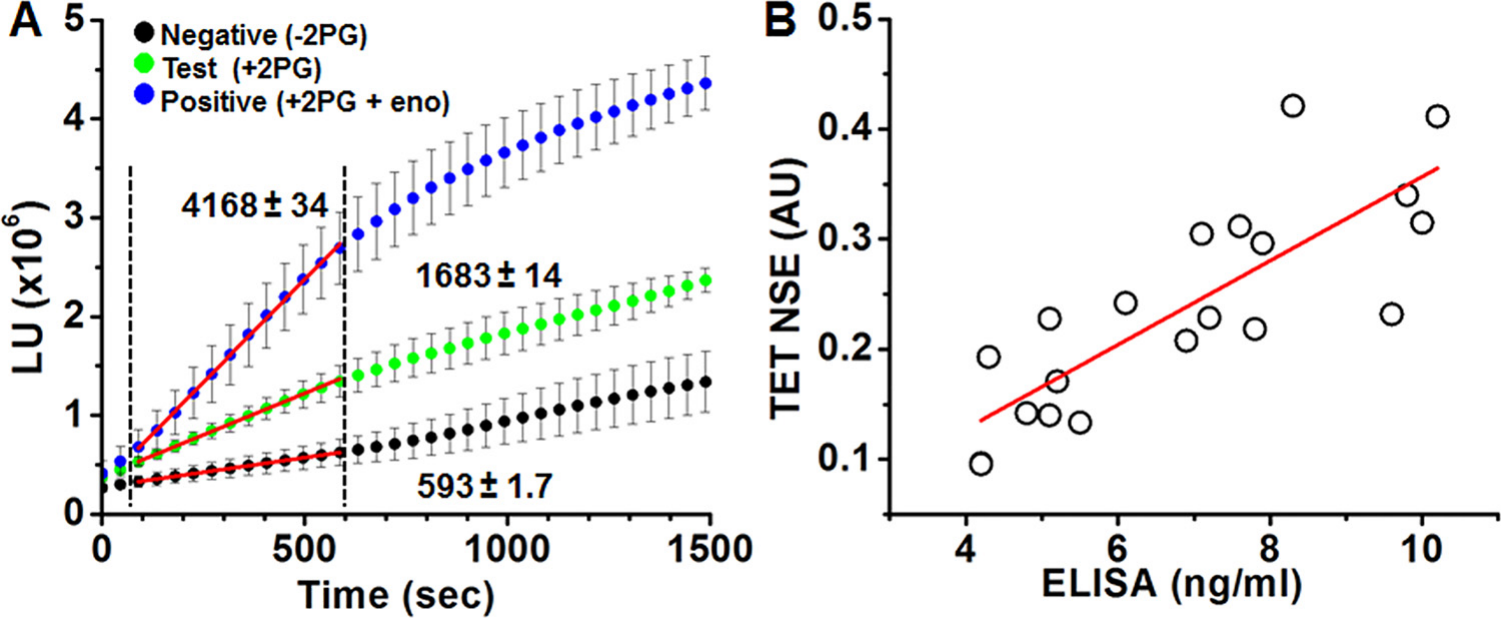
Using the tethered enzyme assay for rapid detection of NSE in human subjects. **A)** Representative data as measured from a total of nine wells per subject (triplicate measurements of negative control with no 2PG; the test sample with 2PG; and positive control wells with 2PG and enolase (Eno); mean±STDEV) For calculation of NSE levels, slopes of averaged curves were calculated for the first 0.5–10 minutes of the enzymatic reactions (indicated by the red linear fit in between the dashed lines), then test wells were normalized to positive and negative wells (see methods section). **B)** High correlation was found between NSE measurements by ELISA (ng/ml) vs. TET assay (AU represents the normalization of test reaction to the negative and positive reactions, as illustrated in A and described in the methods section) as of the human plasma samples. Line indicates best fit. Pearson’s r = 0.815, n = 20.

Moving forward from an animal model, we next tested TET’s ability to detect NSE in the even less defined environment of human plasma. Blood samples were collected from consenting patients seen at the Central New York Alzheimer’s Disease Assistance Center (ADAC) at SUNY Upstate Medical University using Na-Heparin tubes. Plasma was then divided with a portion of the sample tested immediately using TET and the rest frozen at -80°C for later confirmation via commercial ELISA. We ran this experiment using TET in the form of a first generation PoCT technology for human NSE testing. Namely, reactions were designed to work with minimal plasma volume in white 96 well plates in which all test and control reaction components had been lyophilized. 10 μl of freshly collected plasma was added into pre-loaded wells and the light output was measured with a conventional plate reader. TET reaction reading was completed within 10 minutes from the addition of plasma to the pre-loaded wells, where each test included triplicate negative control wells (reaction lacking 2PG), positive control wells (pre-loaded with enolase) and test wells, so that a total of 90 μl of plasma was used for each subject tested (a volume able to be obtained from less than 4 drops of blood). Importantly, using a positive control reaction including known amounts of spiked Eno, allowed us to normalize the data between experiments and minimize any variabilities that might arise from batch to batch differences in enzyme activity. To validate the accuracy of the TET results (representative data shown in Fig 4A), plasma samples were sent for lab testing (ARUP labs, Salt Lake City, UT). Using a Pearson’s correlation test, we compared TET and ELISA data finding a significant correlation of 0.81 between the two approaches (Fig 4B).

## Discussion

Our results suggest that enzymes tethered via oriented immobilization can provide a PoCT platform enabling simple, rapid and objective testing for biomarkers associated with hard to diagnose and time sensitive diseases. We focused on NSE for this proof of concept because it has been shown to convey clinically relevant information for several brain injuries and cancers.

Immobilized enzymes present several important advantages for medical applications including stability and spatial organization in a device, whether with a microfluidic card or paper chromatography. Of note, our data show that oriented enzyme immobilization also significantly improved the efficiency with which coupled enzymatic reactions occurred. This is of special importance because use of coupled enzymatic reactions allows signal amplification at both the stages of biomarker detection and transduction of signal into the luminescent readout. When combined with the inherently enhanced sensitivity and speed of catalytic activity, tethered enzymes provide some of the highly desired features expected from a PoCT diagnostic platform. Demonstrating their suitability for this purpose, we showed in vitro the advantages of using tethered versus soluble enzymes. We showed that TET-based systems could detect physiologically and pathologically relevant concentrations. Importantly, the immobilized enzyme-based detection system provided an ultra-rapid analysis (i.e. within 10 minutes) even when using plasma samples obtained from rat stroke models or human patients.

The high correlation between ELISA and TET suggests circulating NSE enzymatic activity corresponds very well with the amount of protein. This comparison between TET and ELISA in detection of NSE raises another important finding regarding the dynamic range for detection provided by these two methods. Whereas the ELISA method provides a linear detection range, the enzyme-based assay offers close to a 2-fold larger dynamic range (as defined by the difference between the smallest and largest usable signal, and calculated from the max to min values for each assay). Along with the increased assay speed (minutes for TET vs. hours for ELISA), minimal user effort (TET is a “mix and test” assay, while ELISA requires multiple wash steps), this finding revealing the increased sensitivity of TET versus antibody capture techniques provides another feature contributing to the improvements of TET over ELISA.

One potential criticism of use of tethered enzymes is that the range of biomarkers able to be detected through enzymatic reactions represents a limited subset of target analytes. However, there are numerous biomarkers with bona fide enzymatic activity (e.g. nucleoside diphosphate kinase A; NDKA [25], and phosphoglycerate mutase; PGM [26]), or are modulators of enzyme activity (e.g. calcium [27], magnesium [28]), or are substrates and metabolites of enzymatic reactions (e.g. glutamate and glucose [29]). Currently, no single biomarker, including NSE, can accurately and definitively diagnose any disease. Therefore, we are designing and testing TET-based approaches for multiple classes of target analytes, beyond enzymes. This will allow us to simultaneously quantify suites of biomarkers, significantly increasing the accuracy and robustness of the diagnostic test.

Together, our data support further investigations of enzymes tethered to NPs via oriented immobilization in PoCT diagnostics. The advantages of spatial control of enzyme activities, and rapid and sensitive detection with signal amplification at both detection and transduction into luminescence output, combine to make TET a highly attractive alternative to antibody-based detection. These attributes are especially attractive for integrating TET into a PoCT technology for use by paramedics, athletic trainers and in the field. In terms of lack of need for excitation, low power requirements, and potential telemedicine capability, this technology is potentially game-changing for health care management in rural areas and developing countries.

## Materials and Methods

### Ethics Statement

All research involving human participants (including consent forms and procedure) was approved by Cornell (#1410005002) and SUNY Upstate (#623458–5) IRBs. Written and verbal consent were obtained from participating subjects or health care surrogates and documented according to best practices. Samples were given de-identifying codes to ensure participant confidentiality. All animal procedures were reviewed and approved by the Cornell University Institutional Care and Use Committee (#2009–0043) and were conducted in strict accordance with the recommendations in the Guide for the Care and Use of Laboratory Animals (published by NIH).

### Experimental Design

The aim of this study was to explore the advantages of utilizing enzymes tethered via oriented immobilization in the detection of NSE, a neuronal injury biomarker, towards development of a PoCT technology for time sensitive illnesses.

The study was designed to follow 3 successive stages: (i) Test the enzyme-based assay in vitro to examine the effects of oriented enzyme immobilization on the sensitivity and efficiency of the solid phase vs fluid phase reaction. (ii) Test the tethered enzyme assay in plasma from a rat stroke model. Here, to simulate stroke, 2 groups of rats underwent craniectomy under anesthesia, and branches of the MCA were cauterized in the test group. Peripheral blood samples were drawn over a time period of 6 hours to monitor changes in NSE level. Finally, the changes in NSE levels as measured with TET were compared to those obtained with ELISA. (iii) Use pre-filled 96 well plates in a comparative analysis of NSE plasma levels in samples taken from human subjects and measured by both the solid phase TET reaction and ELISA.

### Reagents

Enliten ATP detection kit was purchased from Promega (Madison, WI). 2-PG, PEP, ADP, ATP, luciferin, PK antibody, NSE, and yeast α-enolase were from Sigma (St. Louis, MO). pcDNA4/HisMax TOPO TA vector was from Invitrogen (Carlsbad, CA). Si-NPs (500 nm) were purchased from Spherotech Inc. (Lake Forest, IL).

### Construction of His-Si4 fusion proteins

The complementary deoxyribonucleic acid (cDNA) of PK was obtained by reverse-transcription polymerase chain reaction (RT-PCR) from mouse muscle RNA. The Luc2 sequence was amplified from the Luciferase2 plasmid, Promega (Madison, WI).

His-PK and His-Luc were generated by TA cloning of PK and Luc into pcDNA4/HisMax TOPO TA. To generate the His-Si4 vector, two complementary oligonucleotides (Integrated DNA Technologies, Inc. USA) encoding the Si4 sequence [22] with overhanging A were hybridized and cloned into the TA site of the pcDNA4/HisMax TOPO TA plasmid, followed by restriction/ligation of PCR fragments of PK and Luc to make His-Si4-plasmids. Subsequently, the Si4-PK and Si4-Luc sequences were amplified by PCR and inserted into pcDNA3.1 to make Si4-PK and Si4-Luc. Constructs were validated by sequencing and amplified in TOP10 cells and then purified.

### Protein expression and purification

HEK293-F-FreeStyle cells (Invitrogen, Grand Island, NY) were transfected with plasmids encoding His, His-Si4 or Si4 fusion proteins using the Freestyle MAX reagent (Invitrogen, Grand Island, NY) and incubated for 24–72 hrs in 8% CO_2_. Cells were harvested and lysed by sonication (Sonifier 250, Branson, Danbury, CT). His-tag or His-Si4 fusion proteins were purified on Ni/NTA beads as previously described [30], with the exception that the Si4 tag was used to immobilize PK on silica NPs directly from the cell lysate (S1D and S1E Fig), providing a fast and simpler purification process.

### Enzymatic activity assays

Enzyme activities for both single and coupled reactions were assessed via luminescence output, in sodium phosphate buffer (50 mM) supplemented with MgCl_2_ (5 mM) and KCl (20 mM). The forward reaction for PK is: *PEP* + *ADP* → *pyruvate* + *ATP*. The forward reaction for Luc is: *ATP* + *luciferin* + *Mg* ^2+^ → *hν*. The PK and Luc coupled reaction is: *PEP* + *ADP* → *pyruvate* + *ATP* + *luciferin* → *hν*. All experiments were carried out in 96-well black or white plates at room temperature. Purified or immobilized PK was mixed with Enliten luciferase/luciferin reagent in addition to ADP and PEP as indicated for individual experiments. Purified or immobilized Luc was mixed with luciferin, Mg^2+^, K^+^ and ATP as indicated in individual experiments and activity was monitored by means of light emission as detected by a luminometer (GloMax, Promega, Madison, WI), where the luminescence signal was integrated for 3 seconds every minute for up to 50 minutes, as indicated per each experiment. To assess the activity of the coupled reactions, PK and luciferase were mixed as indicated above, with luciferin, ADP and PEP, and light emission was measured by luminescence. To maximize enzyme activity per NP, protein was added in saturating amounts to the NPs [31].Data processing and analysis were carried out using Excel (Microsoft) and Origin (Origin Lab, Northampton, MA). In experiments with internal repetitions, data are presented as average ± standard deviation. Otherwise, representative experiments are shown that were repeated independently at least three times with similar results.

### Detection of NSE in rat stroke model

#### Surgery and occlusion

Male Sprague-Dawley rats (Charles River, Wilmington, MA) with average weight 400 g (std. dev. 83 g, range 260–530 g) were used in these experiments. Only males were used, to eliminate potentially confounding effects from the stage of estrus cycle. Animals were anesthetized with 5% isoflurane in oxygen and were maintained on 1.3–2.0% isoflurane. Anesthesia was adjusted to maintain both constant breathing rate of ~1 Hz, measured manually, and suppression of pedal withdrawal reflex. Rats were free breathing. In previous work [32], we have found that with these parameters it is not necessary to artificially ventilate animals for consistent cortical blood flow. Body temperature was maintained at 37.5°C with a feedback controlled rectal thermometer. Glycopyrrolate (50 mg/100 g rat) was injected intramuscularly to suppress fluid buildup in the lungs. Rats were placed in a stereotaxic device and incision locations were shaved, cleaned and injected with bupivacaine (0.125%, s.c.) for analgesia. The femoral artery was catheterized with PE 50 tubing for arterial blood draws. The skin and muscle on the right side of the skull were retracted, and a craniectomy was drilled from the temporal bone to just above the zygomatic arch. The dura was reflected. In experimental stroke animals, branches of the MCA were cauterized at both the entry and exit points from the field of view so that there was no flow visible in the exposed branches of the MCA. In sham animals no cauterization was used. The occlusion procedure took approximately 15 minutes, with time 0 recorded at the end of the occlusion procedure. The occlusions were visually checked every 15 minutes to ensure that the clots remained. In a few animals, some vessels became patent within the first hour and were recauterized. Experiments were conducted in matched pairs of sham animals and stroke animals. Blood (0.5 ml) was drawn at 1 hour before occlusion (or sham), and after 0, 1, 3 and 6 hours post occlusion. In order to avoid dilution, flushing of the catheter was minimized. Fluids were replenished by subcutaneous injection of 0.5 ml of 5% glucose after every blood collection.

After the last blood draw, animals were overdosed with pentobarbital. Chest cavities were opened before the heart beat ceased and in some cases blood was drawn from a ventricular cardiac puncture. Animals were intracardially perfused with ~150 ml PBS and 300 mL 4% paraformaldehyde in PBS. Heads were stored in paraformaldehyde. Images were taken of each brain after extraction and before sectioning.

#### Fluoro-Jade C Staining

Brains were harvested and cryoprotected in 30% sucrose and then 60% sucrose for at least 24 hour each. Brains were frozen and cut into coronal, 45-μm sections on a cryotome. For Fluoro-Jade C (FJC; Millipore) staining, sections were washed in 80% EtOH in 1% NaOH for five minutes, 70% EtOH for 2 minutes, and 0.06% KMnO_4_ for 10 minutes. Sections were then incubated in the solution of 0.00015% of FJC solution (1:100 stock, according to manufacturer’s instructions, dissolved in 0.1% of acetic acid) for 20 minutes. Sections were then washed with distilled water 3 times, for 1 minute each, at which time the slides were dried for 5 minutes and put into xylene solution for 5 minutes. Last, slides were dried for an hour and coverslipped with Permount (Electron Microscopy Sciences). Initially, FJC staining was performed at 1 mm-spacing throughout the brain. After the most anterior and posterior sections with FJC staining were identified, additional sections were stained to resolve the stroke volume with 200–300 μm spacing.

#### Measurement of rat brain infarct volume and torn tissue area

Low magnification white light images of the entire section were photographed under a stereoscope. FJC staining was imaged with epifluorescence under 20x magnification using filters for FITC. The area labeled with FJC was manually identified by a person blinded to the treatment and marked on the low magnification, white light images of whole brain sections using ImageJ. For figures, images were normalized by calculating the mean and standard deviation of manually selected background region and then setting the minimum intensity to 4.2 x standard deviation below the mean, and maximum intensity to 7.2 x standard deviation above the mean. Total FJC volume was obtained by multiplying each section’s infarct area by the distance between sections.

In some sections, a part of tissue was torn in the stroke region. To compensate for missing tissue volume, the torn tissue area was obtained by subtracting the area of the ipsilateral from the contralateral side as calculated from the low magnification images. The volume of torn tissue was obtained by multiplying each section’s missing tissue by the distance between sections. The torn tissue volume was then added to the FJC volume to calculate the total stroke volume.

#### Measurements of NSE activity and quantification by ELISA

Plasma was separated from whole blood samples using CAPIJECT Micro Collection Tubes (Terumo Medical Corporation, Somerset NJ). 50 μl samples of plasma were added to individual wells in a 96-well plate, and reaction mix was added just before luminescence measurements were initiated (GloMax, Promega). Reaction mixtures included 1:1 ratio of NP-PK and NP-Luc, with equal mass amounts of enzyme bound to the NPs, resulting in roughly a 2:1 ratio of Luc to PK molecules. To minimize batch-to-batch differences in repeat experiments, the amounts of the NP-PK and the NP-Luc to be added was based on their individual activities. In this way, the relative activities of each enzyme were kept constant among experiments with different batches of protein. Luminescence signal was integrated for 3 seconds, every minute for 50 minutes. Control wells (-2PG) were then subtracted from the raw data and LU were integrated over the first 20 minutes of the measurements. Integrated LU values of each time point were then normalized to t (-1 hour).

Rat NSE was quantified using an ELISA Kit, (R&D Systems, Inc., Minneapolis, MN) according to the manufacturer’s directions. Non-Neuronal Enolase (NNE) was also detected using ELISA Kit, (antibodies-online.com, Atlanta, GA) according to manufacturer’s directions. Assays were read using an Infinite M200Pro microplate reader (Tecan Trading AG, Maennedorf, Switzerland) and Magellan software (Tecan Austria GmbH, Grödig/Salzburg, Austria).

### Detection of NSE in blood samples from human subjects

#### Plasma sample collection

5 ml of blood was collected from enrolled subjects into Na-heparin 6 ml tubes. Tubes were spun at 1000 g for 5 minutes and then plasma was aspirated and placed over ice. Samples were divided and aliquots frozen for later analysis as indicated.

#### Detection of NSE in plasma samples from human subjects

10 μl of freshly collected plasma was diluted with 10 μl of water and added to individual wells of a 96-well plate preloaded with lyophilized TET reagent mixtures for negative, test and positive control wells (in triplicates). The readout luminescence signal was integrated for 0.4 seconds, and read continuously for 25 minutes using a TECAN Safire plate reader. For calculation of NSE levels, the linear regression slope for the initial activity was calculated per each well. Then, control wells (-2PG) were subtracted from the test channel and normalized to the positive control well (with 2-PG and enolase). Positive control reactions allowed us to normalize the results to reduce batch to batch variability. Plasma samples were sent for further analysis to ARUP laboratories (Salt Lake City, UT). Variability for PK and Luc activity between batches was addressed by preparing mixtures of the enzymes with equal activity rather than adding equal amounts of each protein.

## Supporting Information

S1 Fig Construction and expression of PK-affinity tag fusion constructs. **A)** Schematic illustration of fusion PK constructs indicating location of the His and Si4 peptide sequences. Three fusion plasmids were constructed for PK–His-PK, His-Si-PK and Si-PK. **B)** HEK-293 cells were transfected with each of the 3 plasmids. 3 days later, cell lysates were separated by SDS-PAGE and then immunoblotted for PK expression, where His-PK showed highest expression level. **C)** His-PK and His-Si-PK were purified (using the His-tag, see methods section) and tested for their specific activity when not tethered. Protein concentrations were determined with the Micro-BCA assay (Pierce, Rockford, IL), and purity of the samples was analyzed by SDS-PAGE and immunoblotting. **D)** Immunoblot (top) and quantification (bottom) of protein bound to 500 nm SiO_2_ NPs following incubation with whole cell lysates of His-PK, His-Si4-PK or Si4-PK expressing cells. This comparison shows that the Si tag increases >2 fold the amount of protein bound to SiO_2_ NPs. **E)** The activity of His-PK, His-Si4-PK or Si4-PK fusion proteins was measured when immobilized on SiO_2_ NPs following incubation of whole cell lysates with the 500 nm NPs. Oriented immobilization through the Si tag of His-Si-PK (blue squares) and Si-PK (red circles) results in comparable initial reaction rates, while His-PK lacking the SiO_2_ affinity tag reveals a slower activity rate most likely due to its non-specific adsorption to the NPs.
(TIF)

S2 Fig Supplementary analysis for PK-Luc coupled reaction as shown in Fig 1. **A)** PK and Luc coupled activity was assayed in 4 combinations: (NP-PK) + (NP-Luc), (NP-PK) + (soluble Luc), (NP-Luc) + (soluble PK) or (soluble PK + soluble Luc). Luminescence output was measured for each combination with and without PEP, as indicated by the colored symbols. All combinations included equivalent amounts of PK and Luc. Here, coupled efficiency was calculated using the analysis as used in Fig 5, calculated by subtracting the negative reaction (-PEP) from positive reactions (+PEP) slopes (indicated by solid lines). **B)** Summary of data presented in A, shows that having both PK and Luc on NPs (black) facilitates reaction rates compared to other combinations. Each condition was tested in triplicates; data shown represents 3 individual experiments; AVG±STDEV.
(TIF)

S3 Fig Determining the activity of His-Si4-Luc immobilized on silica NP’s. **A)** Schematic representation of experimental setup in the luciferase activity assay. **B)** Representative traces showing the activity of Luc measured when immobilized on silica NPs (blue dots), or in solution (square) with various ATP concentrations. For these experiments, the luminescent signal was normalized against LU at time point 0, and plotted as a function of time, demonstrating a significantly slower decay time for Luc when tethered.
(TIF)

S4 Fig Co-immobilization of PK and Luc on single NPs reduced reaction efficiency when ADP was limiting. **A)** To investigate the effect of enzyme proximity on coupled activity more directly, His-Si-PK and His-Si-Luc were immobilized on 500 nm silica NPs separately (black) or together (red). Luminescence signal in the presence of 0.2, 2 or 6 mM ADP was normalized to t (0 min) and plotted against time (all other conditions/substrates were kept the same). **B)** Surprisingly, we found the activity of the coupled reactions to be significantly reduced when the enzymes were co-tethered on single particles versus tethered on separate particles. There are multiple possible explanations for this finding, ranging from steric hindrance between the two proteins when co-tethered, to interference by Luc with PK tetramers, to a competition between the enzymes for interaction with ADP [33, 34]. Such competition would reduce substrate availability for PK, resulting in overall reduced luminescence. To distinguish between these possibilities, we repeated the experiment with increased concentrations of ADP, and found that a 10-fold excess or more of ADP reversed the reduction in activity (middle and right panels), suggesting that competition for ADP was largely responsible.
(TIF)

S5 Fig Testing various ratios of PK and Luc in coupled reactions to detect Enolase. **A** and **B)** Representative traces showing the luminescence output signal as measured for 3 ratios of PK and Luc on NPs (A) or in solution (B) in coupled reactions to detect Eno (PK:Luc ratios as follows- blue squares- 2:1, red triangles- 1:1, black circles 1:2; filled markers represent reaction with Eno, empty markers represent no Eno). **C** and **D)** average slopes for reaction as presented in A and B (blue bars–no Eno, red bars with Eno, AVG±STDEV). E and F) the calculated signal to background ratio for the 3 different ratios of PK:Luc when tethered **(E)** or in solution **(F**). Each ratio was tested in triplicate; AVG±STDEV.
(TIF)
