# Supplementary figures and images for: Use of Tethered Enzymes as a Platform Technology for Rapid Analyte Detection
Source: PLoS One. 2015 Nov 25;10(11):e0142326. doi: 10.1371/journal.pone.0142326 (PMC4659663; doi:10.1371/journal.pone.0142326)

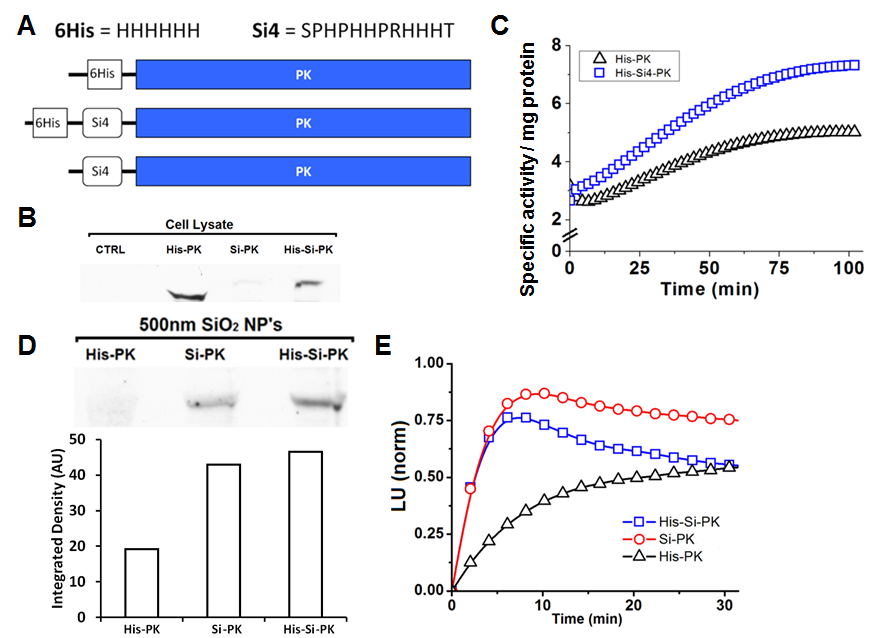

Supplement: S1 Fig — A) Schematic illustration of fusion PK constructs indicating location of the His and Si4 peptide sequences. Three fusion plasmids were constructed for PK–His-PK, His-Si-PK and Si-PK. B) HEK-293 cells were transfected with each of the 3 plasmids. 3 days later, cell lysates were separated by SDS-PAGE and then immunoblotted for PK expression, where His-PK showed highest expression level. C) His-PK and His-Si-PK were purified (using the His-tag, see methods section) and tested for their specific activity when not tethered. Protein concentrations were determined with the Micro-BCA assay (Pierce, Rockford, IL), and purity of the samples was analyzed by SDS-PAGE and immunoblotting. D) Immunoblot (top) and quantification (bottom) of protein bound to 500 nm SiO2 NPs following incubation with whole cell lysates of His-PK, His-Si4-PK or Si4-PK expressing cells. This comparison shows that the Si tag increases >2 fold the amount of protein bound to SiO2 NPs. E) The activity of His-PK, His-Si4-PK or Si4-PK fusion proteins was measured when immobilized on SiO2 NPs following incubation of whole cell lysates with the 500 nm NPs. Oriented immobilization through the Si tag of His-Si-PK (blue squares) and Si-PK (red circles) results in comparable initial reaction rates, while His-PK lacking the SiO2 affinity tag reveals a slower activity rate most likely due to its non-specific adsorption to the NPs. (TIF) [file pone.0142326.s001.tif]

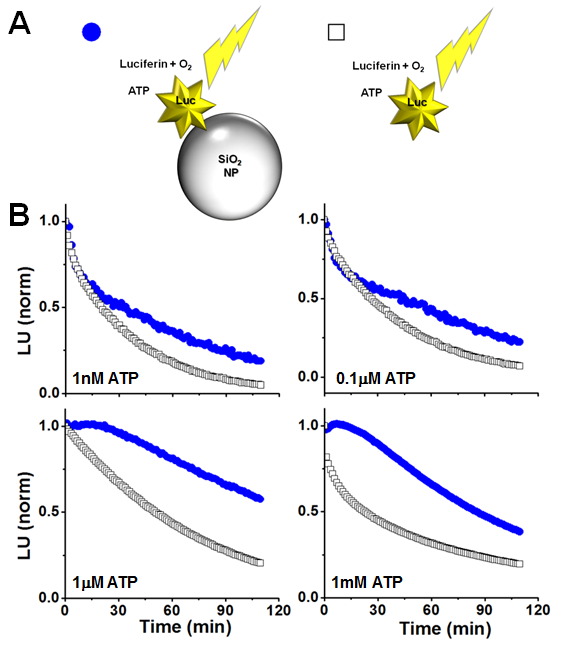

Supplement: S2 Fig — A) PK and Luc coupled activity was assayed in 4 combinations: (NP-PK) + (NP-Luc), (NP-PK) + (soluble Luc), (NP-Luc) + (soluble PK) or (soluble PK + soluble Luc). Luminescence output was measured for each combination with and without PEP, as indicated by the colored symbols. All combinations included equivalent amounts of PK and Luc. Here, coupled efficiency was calculated using the analysis as used in Fig 5, calculated by subtracting the negative reaction (-PEP) from positive reactions (+PEP) slopes (indicated by solid lines). B) Summary of data presented in A, shows that having both PK and Luc on NPs (black) facilitates reaction rates compared to other combinations. Each condition was tested in triplicates; data shown represents 3 individual experiments; AVG±STDEV. (TIF) [file pone.0142326.s002.tif]

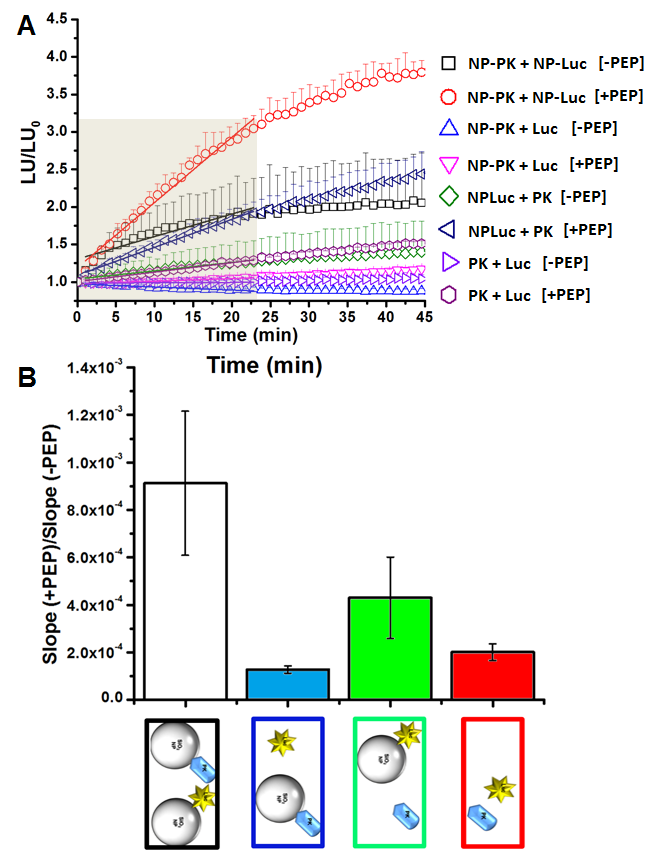

Supplement: S3 Fig — A) Schematic representation of experimental setup in the luciferase activity assay. B) Representative traces showing the activity of Luc measured when immobilized on silica NPs (blue dots), or in solution (square) with various ATP concentrations. For these experiments, the luminescent signal was normalized against LU at time point 0, and plotted as a function of time, demonstrating a significantly slower decay time for Luc when tethered. (TIF) [file pone.0142326.s003.tif]

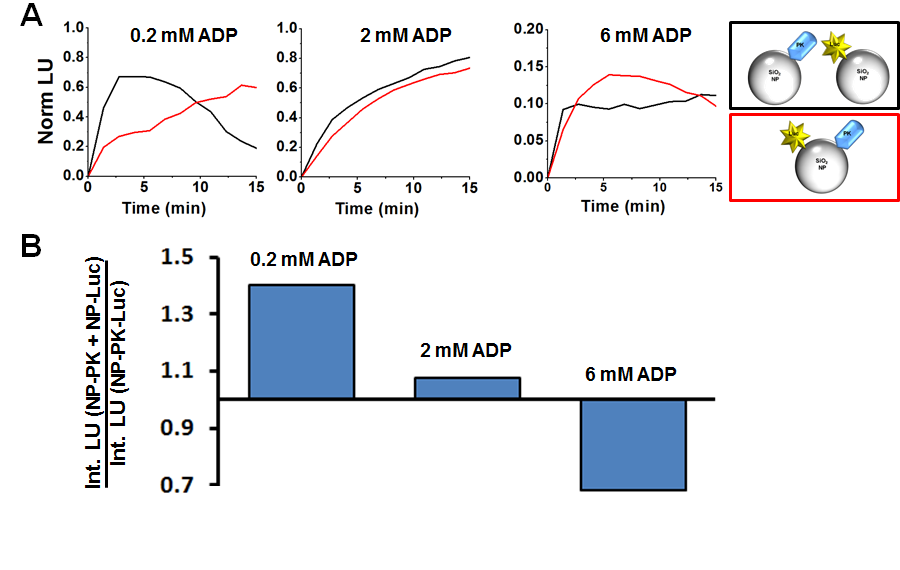

Supplement: S4 Fig — A) To investigate the effect of enzyme proximity on coupled activity more directly, His-Si-PK and His-Si-Luc were immobilized on 500 nm silica NPs separately (black) or together (red). Luminescence signal in the presence of 0.2, 2 or 6 mM ADP was normalized to t (0 min) and plotted against time (all other conditions/substrates were kept the same). B) Surprisingly, we found the activity of the coupled reactions to be significantly reduced when the enzymes were co-tethered on single particles versus tethered on separate particles. There are multiple possible explanations for this finding, ranging from steric hindrance between the two proteins when co-tethered, to interference by Luc with PK tetramers, to a competition between the enzymes for interaction with ADP [33, 34]. Such competition would reduce substrate availability for PK, resulting in overall reduced luminescence. To distinguish between these possibilities, we repeated the experiment with increased concentrations of ADP, and found that a 10-fold excess or more of ADP reversed the reduction in activity (middle and right panels), suggesting that competition for ADP was largely responsible. (TIF) [file pone.0142326.s004.tif]

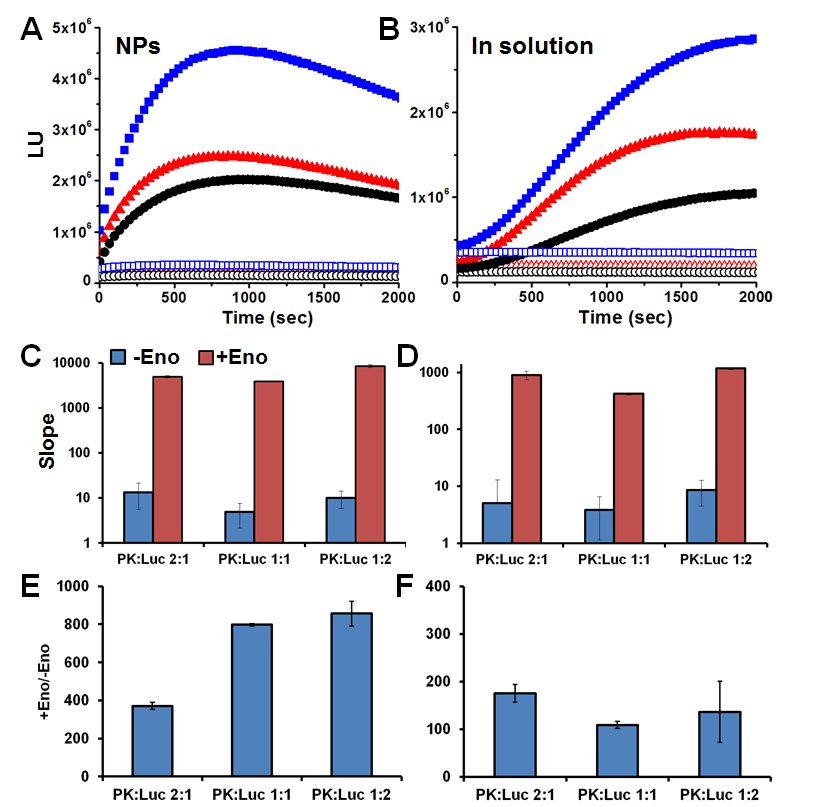

Supplement: S5 Fig — A and B) Representative traces showing the luminescence output signal as measured for 3 ratios of PK and Luc on NPs (A) or in solution (B) in coupled reactions to detect Eno (PK:Luc ratios as follows- blue squares- 2:1, red triangles- 1:1, black circles 1:2; filled markers represent reaction with Eno, empty markers represent no Eno). C and D) average slopes for reaction as presented in A and B (blue bars–no Eno, red bars with Eno, AVG±STDEV). E and F) the calculated signal to background ratio for the 3 different ratios of PK:Luc when tethered (E) or in solution (F). Each ratio was tested in triplicate; AVG±STDEV. (TIF) [file pone.0142326.s005.tif]
